# Efficacy of Antidopaminergic Pharmacotherapy in Pediatric Autoimmune Neuropsychiatric Disorders Associated With Streptococcal Infections (PANDAS): A Case Report

**DOI:** 10.7759/cureus.44164

**Published:** 2023-08-26

**Authors:** Donald Hefelfinger, Hannah Kaufman, Alex Gilman, Rick Gebhart

**Affiliations:** 1 Family Medicine, Wright State University Boonshoft School of Medicine, Dayton, USA; 2 Family Medicine, Gebhart Concierge and Consult, Vandalia, USA

**Keywords:** psychiatry, streptococcal infections, tic disorder, obsessive-compulsive disorder, pandas

## Abstract

PANDAS, or pediatric autoimmune neuropsychiatric disorders associated with streptococcal infections, is a neuropsychiatric disease seen in children that presents with prevailing symptoms of obsessive-compulsive disorder (OCD), tic disorder, or both. These symptoms appear suddenly following a streptococcal infection, such as strep throat or scarlet fever. The antibodies formed to eradicate the streptococcal infection have been shown to alter central dopamine signaling. In spite of being acknowledged in the medical community for the last two to three decades, PANDAS is a disorder that goes unnoticed by many healthcare professionals. Unfortunately, even with correct diagnosis and utilization of commonly prescribed pharmacotherapy and psychotherapy, medical management can often be ineffective at treating the neuropsychiatric symptoms.

Here, we describe a case of a 15-year-old male who presented to the primary care office with complaints of episodic behavioral changes that would occur multiple times daily. The general symptoms were centered around body dysmorphia where the patient became obsessed with fixed portions of food and became highly focused on only specific food types. The symptoms would exacerbate and progress with a dire need to burn calories, leading the patient to damage carpeted areas in the home due to regular and fanatical pacing. The patient underwent trials with serotonergic pharmacotherapy with little to no relief of the psychiatric symptoms. After discussion with his primary care physician, the patient underwent trial management with antidopaminergic therapy that resulted in significant neurological and psychiatric improvement. The use of antidopaminergic pharmacotherapy to target the induction of D1 and D2 dopamine receptors was efficacious in this patient; however, it warrants additional research with a larger sample to determine its effectiveness in the treatment of this psychiatric condition.

## Introduction

PANDAS, or pediatric autoimmune neuropsychiatric disorders associated with streptococcal infections, was first described in a 1998 study and was characterized by the sudden and dramatic onset of obsessive-compulsive disorder (OCD) or tic disorder symptoms [1-6]. It most commonly occurs in children aged 3-12 years following a Group A beta-hemolytic streptococcal (GABHS) infection such as strep throat or scarlet fever. This sudden onset helps to differentiate PANDAS from OCD, which typically develops over months to years [2]. These characteristics are also frequently accompanied by other symptoms such as emotional lability, psychosis, hyperactivity, enuresis, dysgraphia, and deterioration in school performance [1-2]. PANDAS appears to have a predilection for males with a ratio of 2.6 to 1, but this association is not well understood [2].

There are five specific criteria used to diagnose PANDAS: (1) presence of OCD and/or tics, particularly complex or unusual tics, (2) symptoms present between the age of 3 and the onset of puberty, (3) acute onset and relapsing-remitting course, (4) association with GABHS infection and (5) association with neurological abnormalities [1-2,4,7]. It is believed that molecular mimicry plays a role in the pathogenesis of this disease, similar to Sydenham’s chorea, which is associated with rheumatic fever [2-3,8-9]. When infected with GABHS, the body mounts an immune response to proteins on the pathogen. Here, we present a case of PANDAS in a 15-year-old male who responded to treatment with antidopaminergic pharmacotherapy. The interesting features regarding this case were the older age of the patient at initial presentation and his excellent response to antidopaminergic pharmacotherapy as opposed to selective serotonin reuptake inhibitors (SSRIs).

## Case presentation

A 15-year-old Caucasian male presented to the primary care office with his mother with complaints of intermittent behavioral changes that would occur multiple times throughout the day. These symptoms were centered around believing he was overweight and needed to lose weight. Symptoms included the patient refusing to eat certain foods and only eating specific exact portion sizes during each meal. He would walk in circles in his room 100 times to the left and then 100 times to the right due to his obsession with burning calories. This behavior was witnessed during his visit with his primary care physician. He would do this multiple times throughout the day, and it became so frequent that he wore holes through the carpet in his room down to the floorboards beneath. During the several months that this behavior persisted, he lost nearly 25 pounds and stopped doing things that he enjoyed doing. In addition to the described symptoms, the patient also had a flat affect and spoke infrequently.

He had been diagnosed with PANDAS at another hospital just after he turned 15. He had experienced multiple streptococcal pharyngitis infections that were treated with oral amoxicillin. The sore throat and fever would subside after antibiotic treatment but would return around two to three weeks later. These strep throat symptoms were accompanied by the psychiatric symptoms described previously. Prior to the symptom onset, the patient had a BMI of 25.0 and after six months of OCD symptoms, this dropped to 16.0. His weight loss was so extensive that his pediatrician was contemplating inserting a feeding tube. He had no other psychiatric issues prior to the OCD induced by the streptococcal infections. He was in the 50th percentile for height at 69 inches on initial presentation and was a traveling hockey star, being scouted by colleges. Due to the severity of his symptoms, the patient was forced to be homeschooled for a period of five months but was able to continue playing hockey.

After his initial diagnosis, he was trialed on multiple drugs, including sertraline, fluoxetine, paroxetine, and venlafaxine, with little to no relief from the psychiatric symptoms. On presentation to the office, he was started on olanzapine 5 mg. At his one-week follow-up, his symptoms had started to improve. He stopped losing weight, started eating a little more, and walked in circles less frequently. His olanzapine was increased to 7.5 mg but resulted in hypersomnolence. Hence, olanzapine was decreased to 5 mg, and he was started on valproate with the hope for further symptomatic improvement. His dose was started at 500 mg for three days at bedtime and then 500 mg twice daily. His valproate level at the two-week follow-up was found to be subtherapeutic at 35 μg/mL, so his dose was increased to 500 mg three times daily. Upon returning to the office two weeks later, he had gained three to four pounds, was no longer walking in circles in his room, and was back to playing hockey and doing the things he enjoyed.

## Discussion

Much of the debate regarding PANDAS involves reaching the proper diagnosis and determining the most efficacious treatment. Diagnosis is difficult to achieve due to troubles demonstrating a definitive association between streptococcal infections and the acute onset of OCD or tic symptoms [4]. Demonstrating this association requires a significant increase in anti-streptolysin O and anti-DNAse B antibodies that must be monitored over time [2,4]. Another difficulty in diagnosis is the lack of reliable biological markers that accurately identify patients with PANDAS [2]. With many medical providers unfamiliar with the diagnosis and treatment of PANDAS, many children end up seeing numerous specialists before receiving the proper diagnosis. This lack of a definitive diagnosis leads to prolonged neuropsychiatric symptoms and a higher likelihood of adverse outcomes in adulthood. In this case, one exciting aspect of the diagnosis was the patient's age at the onset of symptoms. It has been postulated that PANDAS cases are very uncommon after the age of 12 due to the decline in autoimmune cross-reactivity and the low incidence of GABHS due to the development of antibodies against all strains of the infection [2]. Most patients respond well to antibiotic therapy and would not require more extensive antibiotic prophylactic therapy or psychotherapy [2,6]. However, some patients have persistent neuropsychiatric symptoms and require more extensive therapy.

Treatment of PANDAS is an area of ambiguity as there is a lack of research identifying first-line treatment options. Some proposed treatment options include prophylactic antibiotics, immunomodulators such as intravenous immunoglobulins (IVIGs) or plasmapheresis, SSRIs, cognitive behavioral therapy (CBT), and tonsillectomy [6-8,10]. If left untreated, PANDAS can result in more severe symptoms upon subsequent streptococcal exposure and put children at higher risk of having OCD and tic disorders as adults [2,6]. In this case, the patient not only did not respond to antibiotic therapy but also failed to respond to conventional psychotherapy with SSRIs. He did, however, respond exceedingly well to antidopaminergic pharmacotherapy. With the pathophysiology of this disease involving autoimmune induction of D1 and D2 dopamine receptors, it is understandable why it would respond to a drug targeting this dopamine receptor activation.

The basal ganglia is a set of neurons that lie deep within the cerebral hemisphere and controls motor, limbic, sensory, and associative sets of information through the direct and indirect dopamine pathways [2,11-13]. Thus, the basal ganglia is crucial for appropriate brain function in facilitating proper movement and behavior in healthy human beings [2,12]. Molecular mimicry associated with GABHS infections in which the bacteria emulate the molecular assembly of the host's tissue, launching an autoimmune response, is believed to be implicated in the pathophysiology of PANDAS [2-3,8-9,13]. This autoimmune response prompts the production of antineuronal antibodies that attack host cells rather than the intended streptococcal bacteria [2-3,8-9,11,13]. The antibodies formed have also been shown to induce inhibitory signaling of dopamine D1 and D2 receptors found in the basal ganglia and could subsequently alter the central dopamine pathways [8-10]. Antibody titers for these neuronal antibodies have proven to be associated with the severity and duration of the associated neuropsychiatric manifestations [9,13].

These dopamine pathways in the brain are diverse and affect functions such as behavior, movement, and cognition [12-13]. Antineuronal antibodies are associated with the involuntary movement disorder Sydenham chorea and PANDAS, which are both characterized by the acute onset of tics and/or OCD symptoms. These autoantibodies signal human neuronal cells and activate calcium calmodulin-dependent protein kinase II (CaMKII). In various animal models that were immunized with GABHS antigens, there was significant evidence showing autoantibody production against dopamine receptors that concomitantly alter behaviors and function [12-13]. It is implicated that human monoclonal antibodies isolated from patients with PANDAS target the dopamine D2L receptor (D2R). The expression of human anti-D2R autoantibody V gene in B cells and in the serum of transgenic mice demonstrated that the human autoantibodies target dopaminergic neurons in the basal ganglia and other types of neurons in the cortex [12-13].

## Conclusions

PANDAS is a disease that is not well understood by many physicians in clinical practice making its diagnosis and treatment much more difficult. The lack of evidence-based treatment guidelines and reliable biomarkers creates a therapeutic challenge for physicians. The use of antidopaminergic pharmacotherapy to target the induction of D1 and D2 dopamine receptors was efficacious in this patient and warranted additional research. Further studies must determine definitive treatment guidelines and look at biomarkers to aid the diagnosis. This will help in ensuring a timely diagnosis and treatment for patients and will help minimize the likelihood of neuropsychiatric symptoms persisting into adulthood.
